# The Role of Salivary Diagnostic Techniques in Screening for Active Pulmonary Tuberculosis: A Systematic Review and Meta-Analysis

**DOI:** 10.3390/microorganisms13050973

**Published:** 2025-04-24

**Authors:** Radwan Darwish, Maya Tama, Sidra Sharief, Osama Zeidan, Sara Mohammed Ahmed Rady, Kareeza Selby Chacko, Bindhu Nair, Vijayalakshmi S. Bhojaraja, Jeevan K. Shetty

**Affiliations:** 1 School of Medicine, Royal College of Surgeons in Ireland-Bahrain (RCSI-Bahrain), Busaiteen P.O. Box 15503, Bahrain; 2 Library and Learning Resource Centre, Royal College of Surgeons in Ireland-Bahrain (RCSI-Bahrain), Busaiteen P.O. Box 15503, Bahrain; 3 Department of Anatomy and Biochemistry, Royal College of Surgeons in Ireland-Bahrain-(RCSI-Bahrain), Busaiteen P.O. Box 15503, Bahrain

**Keywords:** tuberculosis, salivary biomarkers, molecular testing, diagnostics, screening

## Abstract

Since the World Health Organization (WHO) issued guidelines for developing a non-sputum test for active tuberculosis (TB) diagnosis that exhibits similar performance characteristics to sputum-based diagnosis, salivary diagnostic techniques have gained prominence as potential screening tools or adjuncts to existing diagnostics. We searched online databases for studies that looked at salivary diagnostic techniques. Afterwards, duplicates were removed, titles and abstracts were screened, and full-text studies were assessed for eligibility based on inclusion and exclusion criteria. The studies chosen for final analysis underwent a rigorous quality assessment following a QUADAS-2 template, and data were extracted. The primary outcome assessed the difference in mean levels of interleukins between TB+ patients and TB-controls (Hedges’ g). We then conducted two subgroup analyses: the first segregated the control group into healthy patients, and those with other respiratory diseases (ORD), and the second addressed three different interleukins separately (IL-6, IL-5, IL-17). The secondary outcome involved comparing salivary molecular diagnostic assays to WHO guidelines. This study is registered with PROSPERO, CRD42024536884. A total of 17 studies, out of an initial 1010, were chosen for the final analysis, but one was then excluded for being of poor quality. Our meta-analyses for the primary outcome revealed minimal diagnostic potential for interleukins. Our first subgroup analysis showed that interleukins were incapable of differentiating active TB patients from both healthy controls and ORD patients. Our second subgroup analysis showed that IL-17 was reduced in active TB patients. Assessment of the secondary outcome revealed that most studies relied on a GeneXpert MTB/RIF assay on saliva, but none fulfilled WHO guidelines for a non-sputum test. Individual biomarkers currently lack sufficient discriminatory power to definitively distinguish active tuberculosis from healthy individuals or those with other respiratory diseases (ORD), reinforcing the need for multi-biomarker panels. Interleukins may be alternatively used as markers for prognosis, severity, or treatment response. Our findings also suggest that assays are unable to meet WHO guidelines.

## 1. Introduction

As it stands, the primary goal of diagnosing TB [1] is, first and foremost, to detect infection, and second, to differentiate between latent TB infection (LTBI) and active TB disease, and this can be achieved using either the tuberculin skin test (TST) or interferon-gamma release assays (IGRA).

TST is performed using the Mantoux technique, which typically involves injecting a small amount of purified protein derivative (PPD) into the skin [2]. This test aims to detect a cell-mediated immune response to TB antigens. If an individual has been previously exposed to TB, their immune system will mount a delayed hypersensitivity reaction within 48 to 72 h, resulting in a raised area of skin known as an induration [2,3]. TST has its limitations, however, including low sensitivity and potential false positives due to antigenic cross-reactivity with the Bacillus Calmette-Guérin (BCG) vaccine and environmental mycobacteria [4].

IGRAs offer a more specific and objective assessment of TB infection compared to TST. Two widely used IGRA tests for TB infection are the T-SPOT^®^.TB and QuantiFERON^®^-TB Gold Plus (QFT-Plus) [5]. The QFT-Plus, a fourth-generation enzyme-linked immunosorbent assay (ELISA)-based IGRA, utilizes antigens that activate both CD8+ and CD4+ T-cell responses. Stimulated by Mycobacterium tuberculosis (Mtb)-specific antigens, these in vitro blood tests measure the release of Interferon-γ (IFN-γ) by T-cells, effectively assessing cell-mediated immune responses [6,7,8].

The WHO initially released recommendations in 2011, approving the use of TST and IGRAs for diagnosing TB infection. Various technologies, including TST, QIAGEN QuantiFERON^®^-TB Gold (QFT-G), QIAGEN QuantiFERON-TB Gold In-Tube (QFT-GIT), and Oxford Immunotec T-SPOT^®^.TB (T-Spot) assays, were evaluated. Subsequently, in 2018, the guidelines were updated to emphasize that TST or IGRAs, or a combination of both, are suitable for diagnosing TB infection. In 2022, the WHO issued specific TB skin tests (TBSTs) recommendations for TB diagnosis. The evaluated technologies included Cy-Tb, Diaskintest^®^, and C-TST (formerly ESAT6-CFP10 test). These tests provide alternative options for diagnosing TB infection, and the WHO Guideline Development Group (GDG) panel in 2022 concluded that TBSTs demonstrate comparable diagnostic accuracy to IGRAs and exhibit superior accuracy to TSTs [9].

The diagnosis of active TB disease also involves the use of imaging modalities—namely, chest radiography, such as X-rays and CT scans—to identify abnormal lung findings that may indicate TB, though lacking specificity [10].

Further, microscopy, specifically sputum smear examination, is a widely available and affordable method but has limitations due to its low sensitivity and dependence on the operator’s proficiency. Sputum culture is considered the gold standard for TB diagnosis, as it involves growing the Mtb from patient samples in a laboratory to identify TB infection [11]. However, due to the long time period associated with mycobacterial cultures, the WHO recommends molecular tests like Xpert MTB/RIF as the first-line diagnostic test for active TB. Xpert MTB/RIF rapidly detects the presence of Mtb while also assessing drug resistance, specifically rifampicin resistance [12].

Unfortunately, there are problems associated with sputum-based diagnosis, given that some patients, such as young children, the severely ill, or those experiencing a dry cough, are unable to produce sputum [13]. Some patients also start coughing during sputum collection, which leads to the spread of aerosols that may facilitate TB airborne transmission [14]. For such reasons, the WHO issued guidelines for the development of a non-sputum test for active TB diagnosis that shows similar diagnostic accuracy to sputum-based diagnosis [13].

Stalwart investigators were quick to recommend using saliva, transferring knowledge from salivary-based molecular testing used for Hepatitis C diagnosis [15], as well as HIV, cardiovascular disease (CVD), and chronic kidney disease (CKD), among other systemic diseases [16]. Saliva also has the advantages of a low protein count, reducing background interference while simplifying sample preparation; convenient and non-invasive collection; and easy storage [17,18,19], despite having a low diagnostic yield for acid-fast bacilli (AFB) when assayed by culture. This problem can be overcome by resorting to molecular testing [20], and so far, the results point towards a paradigm shift in TB diagnostic techniques, as will be demonstrated later in this review.

**Figure 1:**
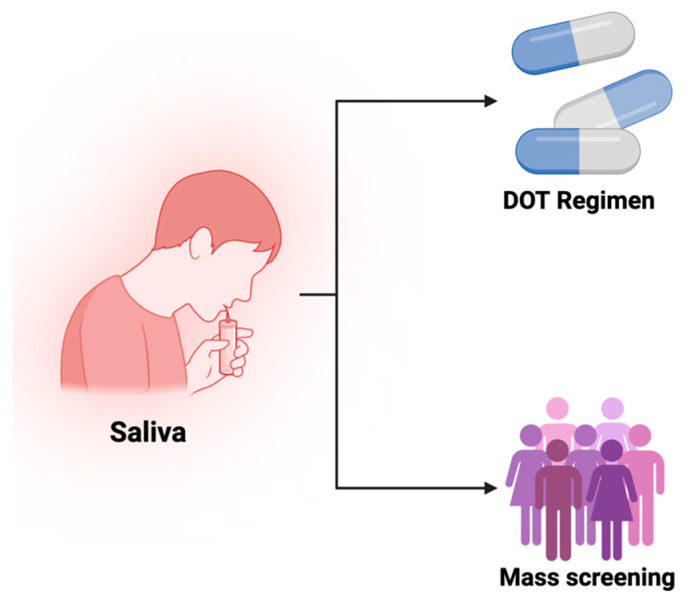
Salivary diagnostic techniques application in Directly Observed Therapy (DOT) regimen and mass screening.

That said, current diagnostic techniques for TB remain invasive, time-consuming, and expensive. As such, we chose to investigate the role of salivary diagnostic techniques as potential screening tools or adjuncts to current diagnostic techniques, as shown in Figure 1 below. We place an emphasis on low- to middle-income countries, where resources are finite.

## 2. Materials and Methods

### 2.1. Search Protocol

A literature search was conducted sequentially across PubMed (380 records), Embase (267 records), Scopus (221 records), Web of Science (136 records), and Google Scholar (6 records) from the inception of each database until 1 April 2024, facilitated by Covidence.

The search strategy utilized Medical Subject Heading (MeSH) terms as shown in the search string below. We focused on identifying English-language studies that discussed the diagnosis of TB using salivary techniques. We also manually explored references from other sources and searched within citations of reviews for any possibly missed articles.

Eligibility criteria included cross-sectional, prospective, and retrospective study designs examining diagnostic accuracy in humans using salivary tests for TB, while exclusion criteria encompassed non-saliva sample studies, animal experiments, reviews, letters, personal opinions, book chapters, theses, conference abstracts, and patents.

### 2.2. Search String

#### 2.2.1. MeSH

((“Tuberculosis/diagnosis”[Mesh] OR “Pulmonary Tuberculosis”[Mesh] OR “Latent Tuberculosis”[Mesh] OR “Tuberculosis, Multidrug-Resistant”[Mesh] OR “Mycobacterium tuberculosis”[Mesh]) OR

(tuberculosis[Title/Abstract] OR “pulmonary tuberculosis”[Title/Abstract] OR “latent tuberculosis”[Title/Abstract] OR “TB”[Title/Abstract] OR “Mycobacterium tuberculosis”[Title/Abstract] OR “MDR-TB”[Title/Abstract] OR “multidrug-resistant TB”[Title/Abstract] OR “drug-resistant tuberculosis”[Title/Abstract])) AND

((“Saliva”[Mesh] OR “Biomarkers”[Mesh] OR “Salivary Biomarkers”[Mesh] OR “Cytokines”[Mesh] OR “Interleukins”[Mesh] OR “Salivary Cytokines”[Mesh] OR “Host-Pathogen Interactions”[Mesh]) OR

(saliva[Title/Abstract] OR “salivary biomarkers”[Title/Abstract] OR “saliva biomarkers”[Title/Abstract] OR “salivary interleukins”[Title/Abstract] OR cytokines[Title/Abstract] OR interleukins[Title/Abstract] OR “host-based biomarkers”[Title/Abstract] OR “immune response markers”[Title/Abstract] OR “salivary cytokines”[Title/Abstract])) AND

((“Molecular Diagnostic Techniques”[Mesh] OR “Nucleic Acid Amplification Techniques”[Mesh] OR “Polymerase Chain Reaction”[Mesh]) OR

(“molecular diagnostic”[Title/Abstract] OR “molecular assays”[Title/Abstract] OR “nucleic acid amplification”[Title/Abstract] OR PCR[Title/Abstract])) AND

((“Diagnosis”[Mesh] OR “Early Diagnosis”[Mesh] OR “Point-of-Care Testing”[Mesh] OR “Sensitivity and Specificity”[Mesh] OR “Predictive Value of Tests”[Mesh]) OR

(diagnosis[Title/Abstract] OR diagnostic[Title/Abstract] OR “point-of-care testing”[Title/Abstract] OR “early detection”[Title/Abstract] OR “predictive testing”[Title/Abstract])))

#### 2.2.2. Non-MeSH

((tuberculosis[Title/Abstract] OR “pulmonary tuberculosis”[Title/Abstract] OR “latent tuberculosis”[Title/Abstract] OR “TB”[Title/Abstract] OR “Mycobacterium tuberculosis”[Title/Abstract] OR “MDR-TB”[Title/Abstract] OR “multidrug-resistant TB”[Title/Abstract] OR “drug-resistant tuberculosis”[Title/Abstract])) AND

(saliva[Title/Abstract] OR “salivary biomarkers”[Title/Abstract] OR “saliva biomarkers”[Title/Abstract] OR “salivary interleukins”[Title/Abstract] OR cytokines[Title/Abstract] OR interleukins[Title/Abstract] OR “host-based biomarkers”[Title/Abstract] OR “immune response markers”[Title/Abstract] OR “salivary cytokines”[Title/Abstract])) AND

(diagnosis[Title/Abstract] OR diagnostic[Title/Abstract] OR “molecular diagnostic”[Title/Abstract] OR “molecular assays”[Title/Abstract] OR “nucleic acid amplification”[Title/Abstract] OR PCR[Title/Abstract] OR “point-of-care testing”[Title/Abstract] OR “early detection”[Title/Abstract] OR “predictive testing”[Title/Abstract]))

### 2.3. Selection of Studies

Two independent reviewers (R.D. and S.R.) examined the titles and abstracts of all studies and eliminated any that were apparently irrelevant to our systematic review, or that followed study designs not included in our eligibility criteria, predominantly reviews and conference posters. A third independent reviewer (O.Z.) then resolved any conflicts that arose. Further, two independent reviewers (R.D. and S.S.) screened full texts. A third independent reviewer (M.T.) then resolved any conflicts that arose. Table 1 shows our eligibility criteria for (a) primary outcome and (b) secondary outcome.

Finally, a quality assessment was performed independently by two reviewers (R.D. and K.C.) using the Quality Assessment of Diagnostic Accuracy Studies (QUADAS-2) tool, which determines the risk of bias by assessing each study against four domains: patient selection, index test, reference standard, and flow and timing of the study. Each domain was addressed through questions that assess both bias and applicability, with responses rated as “high”, “low”, or “unclear”. Inter-rater agreement between two independent reviewers (M.T. and S.S.) was high, and consensus was achieved by consulting a third reviewer (R.D.), who made an unbiased decision regarding conflicted studies.

### 2.4. Protocol for Data Extraction

We selected 17 articles for the systematic review, and two independent researchers then used a standardized data extraction template to collect data from the studies. This template included (1) general information (study ID, title, authors, country), (2) methods (aim, study design, start date, end date), (3) participants (mean age, total sample size, healthy sample size, TB+ sample size, ORD+ sample size, inclusion and exclusion criteria), and finally (4) data relating to the outcome (salivary biomarker levels, salivary assays vs. gold standard).

The studies were then segregated into two categories, the first involving salivary biomarkers, and the second molecular diagnostic assays, and were accordingly used to address the primary and secondary outcomes of our review, respectively.

### 2.5. Data Analysis

We analyzed quantitative data regarding salivary biomarker levels using random-effects meta-analyses that utilized the Hedges’ g statistic with associated 95% CI. This allowed us to estimate the effect size for the difference between the mean levels of interleukins (IL) between TB+ patients and TB- controls.

Using the metafor package on R [1], we devised a meta-analysis that looked at interleukin (IL-1Alpha, IL-1Beta, IL-2, IL-5, IL-6, IL-8, IL-9, IL-12p40, IL-16, IL-17, IL-23) levels in TB+ patients and TB- controls. Missing standard deviations [21] were roughly calculated using the interquartile range (IQR), where SD = IQR/1.35 as per the Cochrane protocol. We then plotted a funnel plot that examined small-study effects and publication bias. To verify our results, we then ran an Egger’s test.

We then conducted two subgroup analyses. The first analysis separated the control groups into healthy controls, and patients with ORD. These were conducted separately, and random-effects forest plots were plotted accordingly. The second analysis included three a priori forest plots, each looking at one of the interleukin levels that were reported in 3 or more samples: IL-6 (n = 5), IL-5 (n = 3), IL-17 (n = 3).

## 3. Results

### 3.1. Article Screening Process

The search strategy described in the previous section yielded 1010 studies, of which 360 were duplicates, detected automatically and manually. Another 592 studies were excluded after title and abstract screening, deemed irrelevant to our review. Full texts of a total of 58 studies were then screened, of which 41 studies were then excluded for having irrelevant outcomes, indications, interventions, study designs, patient populations, and so forth. Articles with unavailable full texts were also excluded because they could not be accessed. The quality assessment then led to one study being excluded for being of poor quality, meaning that 16 studies were included in the final analysis. The excluded study had a small sample size, which could lead to sampling error and provide deviant results from the true population. The entire quality assessment is depicted in Table S1, in the Supplementary Materials.

**Figure 2:**
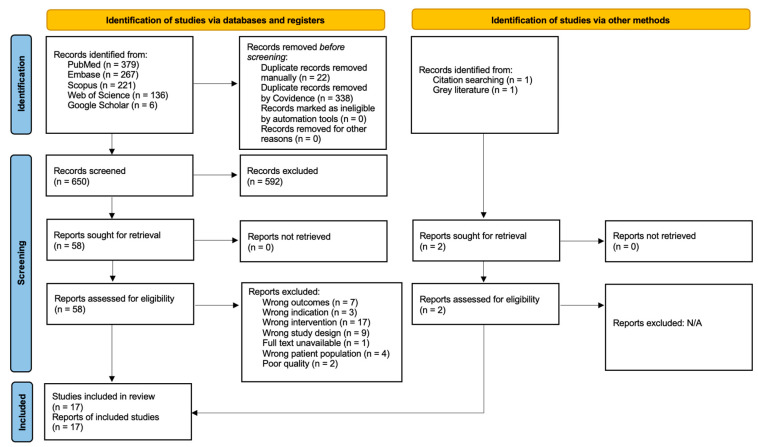
PRISMA Flowchart.

The study selection process is transparently documented using the Preferred Reporting Items for Systematic Reviews and Meta-Analyses (PRISMA) flowchart, shown in Figure 2. This study is registered with PROSPERO, CRD42024536884.

### 3.2. Characteristics of Selected Studies

The selected studies provided baseline data for 2046 participants, divided among two categories of articles: salivary biomarkers (n = 6), and molecular diagnostic assays (n = 10). The characteristics of these studies are further discussed in Tables S2 and S3, in the Supplementary Materials.

Studies investigating salivary biomarkers followed similar steps of salivary collection into test tubes, transportation at 4–8 °C, centrifugation, then storage at −80 °C until use. Transportation and storage at low temperatures aimed to prevent protein degradation, but Estevez et al. [22] additionally treated the samples with protease inhibitors to further promote their stability. Most biomarker studies [21,22,23,24] used multiplex immunoassays such as the Bio-Plex platform (Bio-Rad, Hercules, CA, USA) to measure multiple cytokines simultaneously. Estevez [22] et al. used MagPix (Luminex, Austin, TX, USA), a slightly different multiplex immunoassay device. Pradeep et al. [23] used enzyme-linked immunosorbent assay (ELISA) kits from different manufacturers, utilizing one kit for the measurement of each cytokine separately. To mitigate potential bias, all included studies implemented blinded procedures during the measurement of biomarkers.

Important characteristics of studies included in the meta-analysis are included in Table 2 below.

### 3.3. Primary Meta-Analysis

**Figure 3:**
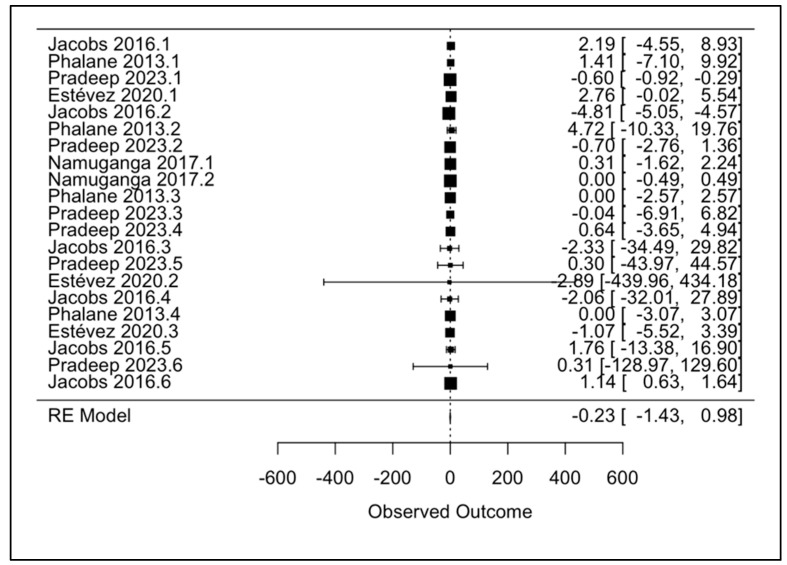
Random-effects forest plot examining the mean difference between levels of interleukins in TB+ patients and TB- controls (including Jacobs 2016.2) [21,22,23,24,25,26].

We conducted a priori meta-analysis of the mean difference in interleukin levels (pg/mL) between active TB patients and TB- controls (healthy or ORD). The interleukins and their corresponding levels are described in Table 3 below. After conducting a leave-one-out analysis, shown in Table S4 (Supplemental Materials), Jacobs 2016.2 was excluded due to its effect size of −4.81 having a large influence on the results, specifically heterogeneity (I2) and variance (Tau2). Figure 3 shows a random-effects forest plot, excluding Jacobs 2016.2.

**Figure 4:**
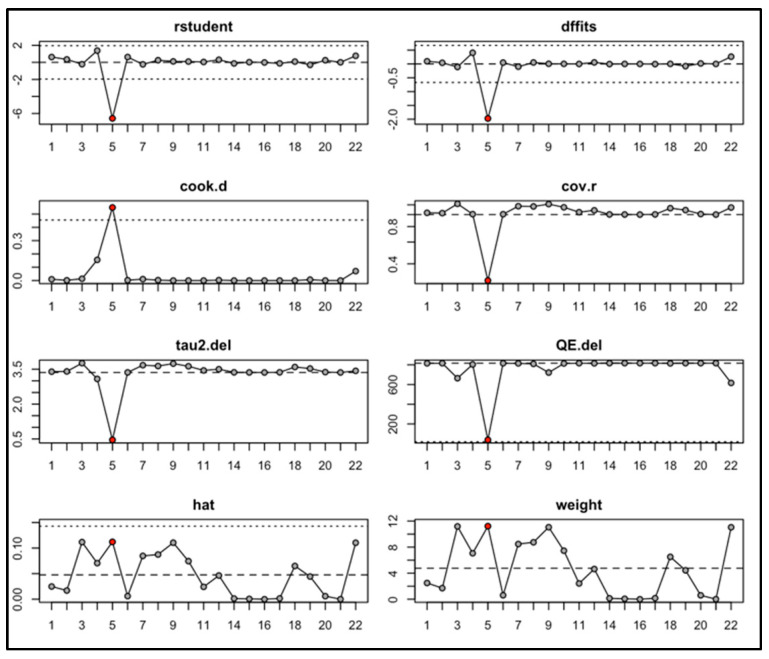
Diagnostic influence and sensitivity analysis for identifying outlier and influential studies in meta-analysis. Visualization of (*Y*-axis) studentized residuals (rstudent), difference in fits (DFFITS), Cook’s distance (cook.d), covariance ratios (cov.r), tau-squared deletion (tau2.del), Q-statistic deletion (QE.del), hat values (hat), and study weights (weight) across (*X*-axis) study or observation numbers (1–22).

Influence diagnostic plots were also created to help visualize the effect of the Jacobs 2016.2 sample on different diagnostic parameters that included model fit, variance, and precision. Figure 4 shows that several studies exceeded conventional thresholds (indicated by dotted lines), and those marked in red were flagged as potentially influential. Rstudent displays studentized residuals for each study, falling below any acceptable range for Jacobs 2016.2. dffits found a large negative influence of the sample on the overall results. It also recorded a large Cook’s distance, which shows that removing the sample significantly affects the results. Cov.r, or covariance ratio, shows the effect of removing the study on the precision of the meta-analysis and drops significantly when the sample is removed. Tau2.del looks at variance between the studies, or heterogeneity, while QE.del displays the effects of removing each sample on residual heterogeneity, and they both fall when the sample is excluded. Hat values are used as indices of leverage, and though it was not as significantly elevated as other parameters for the Jacobs 2016.2 sample, it still has a relatively high leverage contributing to fitted values. Finally, weight refers to the weight of each study as part of the meta-analysis, and the results were similar to those for the hat parameter.

Further, the random-effects forest plot shows a combined negative effect size of −0.23 (95% CI: −1.43–0.98), with confidence intervals that include 0, and a *p*-value of 0.7134. There is no clear evidence of a statistically significant effect, implying that interleukins might not be suitable diagnostic biomarkers for TB.

**Figure 5:**
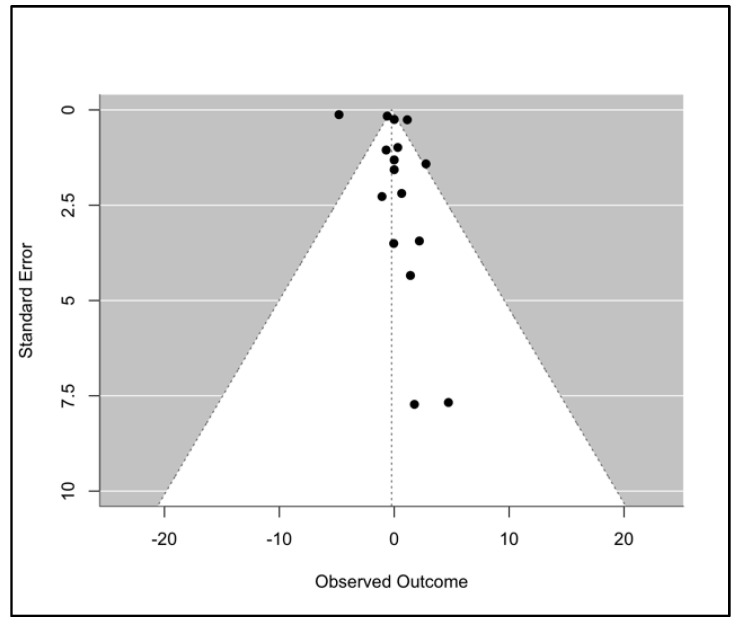
Funnel Plot.

We then constructed a funnel plot to assess publication bias, which was generally symmetrical, as shown in Figure 5 below. Still, many outliers were found within the white areas, suggesting heterogeneity or potential publication bias in the studies. Also, not all studies are shown by the funnel plot, because of dimension restrictions, with two exhibiting very high standard errors. To evaluate the degree of publication bias, we ran Egger’s test. The results are summarized in Table 4 below.

These reveal that while the intercept has a value of −2.7160, indicating the presence of bias, the *p*-value was 0.2335 (>0.05), meaning there is no statistically significant evidence of publication bias or funnel plot asymmetry.

### 3.4. Subgroup Analyses

Previous studies suggest that interleukins are elevated in a plethora of respiratory diseases, including chronic obstructive pulmonary disease (COPD), asthma, and pulmonary fibrosis, especially during exacerbations. Our most studied marker, IL-6, is often implicated in such diseases.

**Figure 6:**
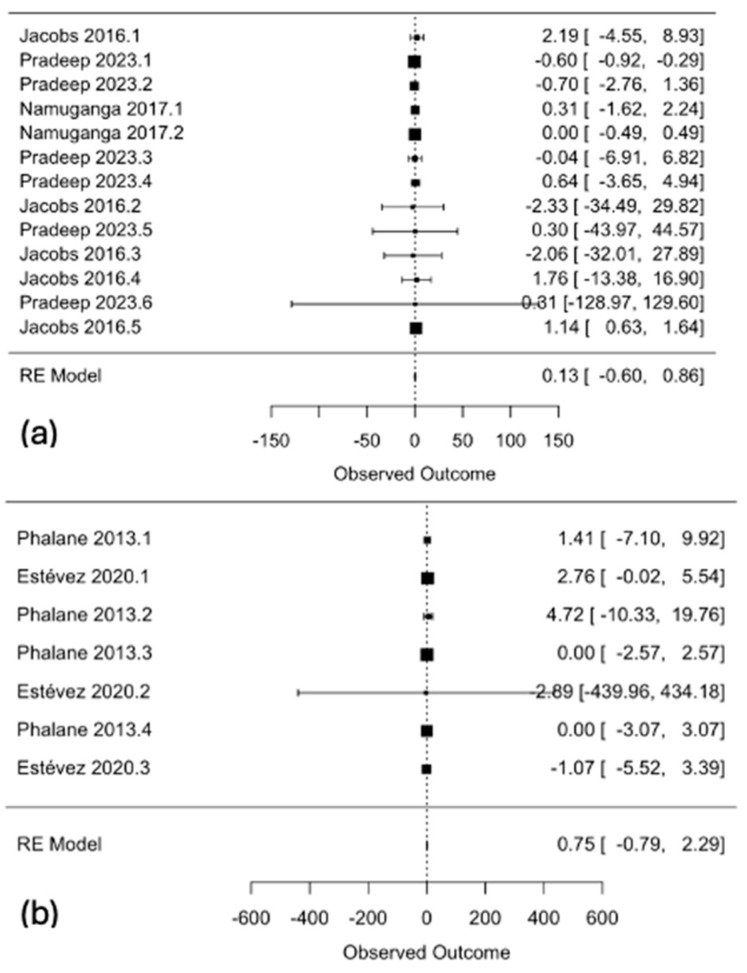
Random-effects forest plot comparing (**a**) ORD patients with TB+ patients and (**b**) healthy controls with TB+ patients [21,22,23,24,25,26].

Accordingly, we conducted a subgroup analysis using Hedges’ g with associated 95% CI to account for upward bias in Cohen’s d, splitting the control group into two categories: those classified with ORD and healthy controls. We then constructed two random-effects forest plots (Figure 6), shown sequentially as (a) for ORD and (b) for healthy controls.

The random-effects forest plot comparing ORD patients with TB+ patients shows a combined negative effect size of 0.13 (95% CI: −0.60–0.86), with confidence intervals that include 0, and a *p*-value of 0.7230. Meanwhile, the random-effects forest plot comparing healthy controls with TB+ patients shows a combined positive effect size of 0.75 (95% CI: −0.79–2.29), with confidence intervals that include 0, and a *p*-value of 0.3381. There is no clear evidence of a statistically significant effect, implying that interleukins might not be suitable diagnostic biomarkers for TB. However, the combined negative effect size in the forest plot (ORD/TB+) fits our speculation that interleukins, which are inflammatory markers, are elevated in ORD.

**Figure 7:**
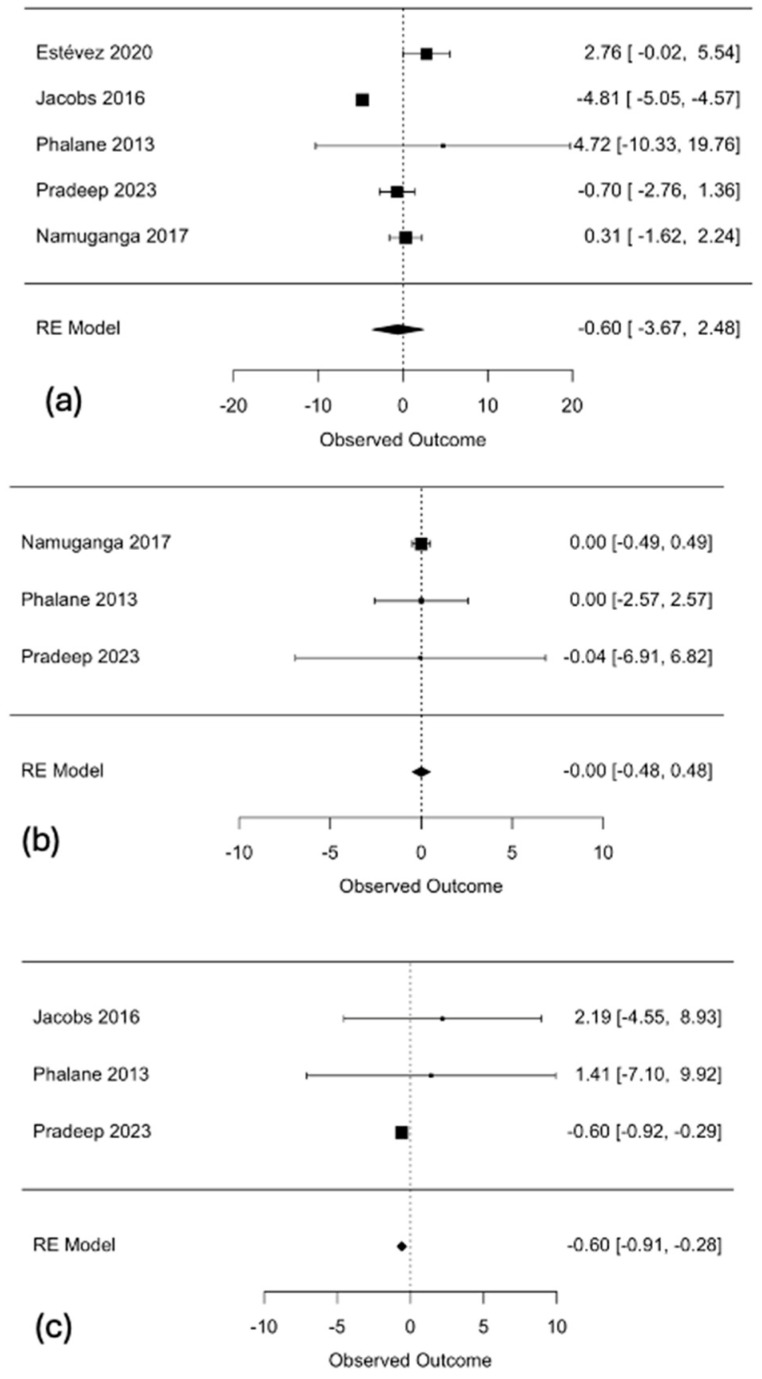
Random-effects forest plots comparing (**a**) IL-6 (**b**) IL-5, and (**c**) IL-17 between TB+ patients and TB- controls [21,22,23,24,25,26].

However, although interleukins are known inflammatory markers, they belong to a broad class of cytokines that carry out various functions throughout the body. To account for these changes, we carried out a second subgroup analysis for all interleukins that were studied three or more times, and these were IL-6 (n = 5), IL-5 (n = 3), and IL-17 (n = 3). Again, random-effects forest plots were constructed and are displayed in three sequential parts, as shown in Figure 7 below.

The random-effects forest plot comparing IL-6 levels among TB+ patients and TB- controls shows a combined negative effect size of −0.60 (95% CI: −3.67–2.48), with confidence intervals that include 0, and a *p*-value of 0.7034. The random-effects forest plot comparing IL-5 levels among TB+ patients and TB- controls shows a combined negative effect size of 0.00 (95% CI: −0.48–0.48), with confidence intervals that include 0, and *p*-value of 0.9993. The random-effects forest plot comparing IL-17 levels among TB+ patients and TB- controls shows a combined negative effect size of −0.60 (95% CI: −0.91–−0.28), with confidence intervals that do not pass through 0, and a p-value of 0.0002. There is no clear evidence of a statistically significant effect for IL-6 and IL-5, indicating that interleukins might not be suitable diagnostic biomarkers for TB. However, IL-17 levels were significantly lower among healthy controls, when compared to TB+ patients.

### 3.5. Salivary Molecular Diagnostic Assays

The remaining 10 articles discussed different salivary molecular diagnostic assays that can be used to detect Mtb. We looked at the assay each one used, sensitivity (%), specificity (%), area under the curve [27], and *p*-value, and then compared them to minimal target product profiles for triage as per WHO standards, detailed in their guidelines for the development of a non-sputum test for active TB diagnosis [13].

As for studies utilizing molecular assays, they included GeneXpert MTB/RIF (Classic and Ultra), direct smear for AFB, auramine rhodamine staining, salivary sIg-A response, lateral flow assay, and Lipobiotin-capture Magnetic Bead Assay. Sensitivity varied greatly, with the lowest sensitivity being 38% with GeneXpert MTB/RIF [28] and the greatest 100% with the lateral flow assay. As for specificity, it varied from 26.25% with GeneXpert MTB/RIF [28] to 100% with GeneXpert Ultra [29]. While all assays met the goal and potential market for triage, none met the minimal target product profile (TPP) for triage, with few studies meeting target specificity and even fewer fulfilling target sensitivity.

The criteria for community-based triaging we chose are detailed in Table 5 below, with the entire comparison displayed in Table S3 as part of the Supplementary Materials.

Generally, the most used assay was GeneXpert MTB/RIF (Ultra and Xpert), and most studies using it met the goal, potential market, and diagnostic specificity criteria. However, only one study [30] met the criteria for diagnostic sensitivity, and none for price.

## 4. Discussion

### 4.1. Interleukins and Tuberculosis

While culture remains the gold standard for the diagnosis of active pulmonary TB due to its high specificity, it is time-consuming, resource-intensive, and requires biosafety level 3 (BSL-3) laboratory infrastructure [31,32]. Therefore, we propose salivary biomarkers as adjuncts to diagnosis enabling cheaper and faster point-of-care screening, especially in resource-limited settings [26]. Although culture can take several weeks to yield results, salivary biomarker assays, based on host immune responses, can provide results within hours [26].

In our meta-analysis, we explored the salivary levels of IL-1α, IL-1β, IL-2, IL-5, IL-6, IL-8, IL-9, IL-12p40, IL-16, IL-17, and IL-23 in TB+ patients and TB- controls. Historically, evidence has implicated interleukins in mediating inflammation and erroneous immune responses in TB. As such, their potential use as diagnostic serum biomarkers was proposed and explored extensively in the literature.

#### 4.1.1. IL-6

IL-6 is an important cytokine that accumulates in TB through other cytokines, including but not limited to Tumor necrosis factor-α (TNF-α). It is produced by both monocyte-derived and recruited macrophages, where Mtb exploits the induction of IL-6 to inhibit IFN-γ-induced autophagy and evade the innate immune response [33]. Members of the suppressor of cytokine signaling (SOCS) family, namely SOCS3 and SOCS1, have also been implicated in the regulation of IL-6 [34,35,36]. IL-6 would be elevated in TB patients, compared to healthy/ORD patients. However, our meta-analysis for IL-6 across the included studies revealed a pooled effect size of 0.60 (95% CI: −3.67 to 2.48).

It might be more prudent to use IL-6 as a biomarker of disease severity or treatment efficacy, as has been demonstrated in previous studies, albeit often in combination with other biomarkers [37,38,39,40,41,42,43,44]. It is thought that the decrease in IL-6 levels indicates recovery during active TB [37], but inter-subject variability remains high, owing to the multifaceted nature of the cytokine. Further, IL-6 signaling is postulated to occur predominantly through the janus kinase/signal transducer and activator of transcription (JAK/STAT) pathway, specifically via STAT3, inducing the release of acute phase proteins, in turn leading to the activation of T-, B-, and myeloid cells [45,46,47]. Acute-phase protein release can also be induced via rapidly accelerated fibrosarcoma (RAF), rat sarcoma (RAS), and mitogen-activated protein kinases, culminating in the same pro-inflammatory environment [48].

Additionally, one genome-wide association study (GWAS) of circulating serum IL-6 levels identified novel loci implicating IL-6 in several inflammatory and immunological pathways [27]. That said, IL-6, as a global indicator of inflammation, may also be elevated in ORDs [49,50]. Therefore, IL-6 should be explored as a prognostic marker for TB, preferably in combination with other biomarkers, noting the possible high false positive rate it may yield.

#### 4.1.2. IL-5

IL-5 is primarily known for its role in eosinophil activation and promoting antibody production by B-cells [51,52]. Previous evidence suggests that IL-5, classically released from T-helper 2 (Th2) cells, assists in reducing Th1 cell responses specific to Mtb in co-infected individuals. It would naturally be expected that the production of both TNF and IFN-γ by monocytes would decrease; however, this has only been demonstrated for TNF. One purported theory speculates that TNF production precedes that of IFN-γ upon cluster of differentiation (CD)4+ and CD8+ stimulation, such that IL-5 modulates the production of IFN-γ over a longer period [53,54]. IL-5 has also been hypothesized to alternatively activate macrophages to suppress Th1 cells in order to control and reduce Mtb growth, though not as readily and effectively as classically activated macrophages [55,56,57]. Other lines of evidence suggest that IL-5 reduces the release of pro-inflammatory cytokines and silences Th1 cell activity by inducing the expression of nitric oxide (NO) synthase within infected macrophages [58,59,60], an observation made by van der Veen and colleagues on microglial cells belonging to a myeloid lineage [58].

Taken together, the literature suggests a role for IL-5 in controlling TB infection and reducing disease severity, though speculation remains rampant. Our results indicate a pooled effect size of 0.00 (95% CI: −0.48 to 0.48). Therefore, IL-5 salivary levels should alternatively be explored to measure responses to treatment.

#### 4.1.3. IL-17

IL-17 is yet another pro-inflammatory cytokine that induces chemokine gradients in response to intracellular pathogens that include Mtb [61,62,63]. It is the main effector cytokine of Th17 cells, classically controlled by RAR-related orphan receptor gamma (RORγt), a transcription factor [64]. During mycobacterial infection, IL-17 mediates the accumulation of both mononuclear and polymorphic cells, while also increasing their infiltration into lung tissue, as demonstrated in a BCG model [65].

Evidence suggests that IL-17 protects neutrophils from death in granulomas, hence delaying recrudescence [66,67]. Some studies also suggest that increased Th17 cell populations increase mononuclear inflammation during initiation of TB infection, leading to earlier death in animal models [68,69,70]. This implies the existence of a bi-directional relationship between Th1 and Th17, where each balances the inflammation of its counterpart to promote survival [71]. The pathways connecting IL-17, among other interleukins, to T-helper cells are complex and hard to navigate. It would, however, be safe to deduce that IL-17 has diverse functions in mycobacterial infection and so would not be very predictive of the presence of Mtb.

Our findings indicate that IL-17 levels are reduced in TB+ patients, with a pooled effect size of −0.60 (95% CI: −0.91–−0.28). These could not be fully explored using the existing literature, given the multifactorial nature of IL-17. Also, these results must be interpreted with caution given that the sample size was small, preparation methods varied, and the results were heterogenous (due to different magnitudes of positive association).

That said, it would not be appropriate to discuss IL-17 without mentioning IL-23, another player in inflammation responsible for maintaining Th17 cells. Together, they help clear intracellular microorganisms, like Mtb, using mechanisms similar to those explained above [71,72,73,74,75]. However, this intimate relationship has not been fully explored and much remains to be discovered. Therefore, IL-17 can be used, possibly in combination with IL-23, to measure the strength of immune responses, informing future treatment plans. These findings can be further validated by comparing levels to IGRAs and blood transcriptomes, among other tests that can help measure and quantify immune responses.

#### 4.1.4. The Role of Interleukins in Latent Tuberculosis

First, IL-6 levels were elevated in patients with active TB, when compared to those with LTBI [22], as shown in Table 2 above. This belies its role in sustaining a pro-inflammatory environment that aids the survival and growth of TB [40]. Reduced levels during LTBI are consistent with the characteristic immune containment of Mtb.

Further, IL-5, which is usually associated with Th2 responses, has been reported to be greater in the serum of patients infected with latent TB, though not consistently [76]. One study, conducted in 2020, examined the systemic level of serum IL-5 in active pulmonary TB, and its alteration across different stages of treatment. The findings indicated that IL-5 is systemically reduced in patients with active pulmonary TB, unlike LTBI and healthy controls. This implies that Mtb might possess a specialized mechanism that allows it to subvert the anti-inflammatory effects of IL-5, ultimately leading to unchecked inflammation in active TB. The same study also revealed a further reduction in IL-5 in post-treatment patients, supporting our hypothesis that Mtb-driven downregulation of IL-5 contributes to disease pathogenesis and is not solely a consequence of bacterial burden [77].

IL-17 would also be expected to fall in patients latently infected with TB. This aligns with its role in delaying recrudescence by protecting neutrophils from death in granulomas [66,67] while increasing mononuclear inflammation [68,69,70]. The same principles can be transferred to IL-23, also expected to fall during latency, and for similar reasons [67]. That said, patients with LTBI are underrepresented in the selected studies, and these findings cannot be extrapolated if not confirmed by more diagnostic test studies.

### 4.2. Molecular Diagnostic Assays

The molecular diagnostic assays for detecting Mtb in saliva included GeneXpert MTB/RIF (Xpert), GeneXpert MTB/RIF Ultra, auramine rhodamine staining, salivary sIg-A response, lateral flow assay using polyclonal antibody against Ag38, and lipobiotin-capture magnetic bead assay. The full comparison to WHO Triage TPP is displayed in Table S3.

Lateral flow assay using polyclonal antibody against Ag38 and auramine rhodamine staining exhibit promising diagnostic sensitivities, but poor specificities, limiting their use in a clinical setting for triage. Further, GeneXpert assays show the best diagnostic performance overall, but the heterogeneity in results between studies mandates standardized testing across different settings and populations. It also seems that these assays are not economically feasible according to WHO TPP guidelines, with a price of around USD 7.97 per unit, meaning that their use in low-resource settings is limited unless subsidized by local or international organizations or governments.

The WHO has previously pointed to GeneXpert as the option of choice for rapid and simultaneous detection of TB and rifampicin resistance in serum [12]. However, the same findings do not transfer well to salivary samples. None of the assays we discussed met the main criteria set by the WHO.

Lateral flow assay using polyclonal antibody against Ag38 and auramine rhodamine staining exhibit promising diagnostic sensitivities, but poor specificities. However, lateral flow assay, specifically, can be revisited, as perfect sensitivity is reported (100%), and poor specificity is reported (42.86%). This test can be used as first-line screening in a community setting, flagging potential cases that would be later reviewed using a centralized GeneXpert assay. This sequential strategy could be a feasible approach, considering we are still far from creating a gold-standard salivary diagnostic assay.

### 4.3. Limitations

Though insightful, our initial primary meta-analysis considered all interleukin levels without distinguishing between classes. Historically, some interleukins have been associated with inflammatory states, and others with protective functions. The heterogeneity of functions served throughout the body led to heterogeneity in salivary levels among TB+ patients. It would also be interesting to learn how administration of the BCG vaccine can affect interleukin levels. To the best of our knowledge, there have been no studies on the relationship between the BCG vaccine and salivary interleukin levels.

Further, our meta-analysis did not distinguish between different variants of TB (active and latent), nor did it differentiate between different stages of TB or stages of treatment. Seeing how labile interleukins can be, this could have led to changes in our data. However, the clinical environment is a diverse one, with TB+ patients presenting at different ages, with different strains, treatment regimens, adherence, and so forth. Therefore, a practical salivary biomarker diagnostic test must perform well across all these categories to be considered for community triage.

Further, it would have been beneficial to explore salivary biomarkers beyond interleukins; however, due to limited studies and limited data, this could not be conducted. Some biomarkers that proved potentially diagnostic in serum include vascular endothelial growth factor levels (VEGF) [78], and transforming growth factor beta (TGF-β) [79], both intimately connected to TB pathogenesis and virulence.

Also, the collection and handling of saliva from individuals suspected of having active TB poses significant biosafety concerns. Though Mtb loads in saliva may be lower than that in sputum, viable bacilli may still be disseminated during sample processing steps that generate aerosols [80]. This is especially pertinent when using molecular diagnostic platforms such as GeneXpert MTB/RIF, which involve mechanical lysis and amplification steps that may pose an occupational hazard without proper containment. Therefore, strict adherence to biosafety level 3 (BSL-3) practices, including the use of certified biosafety cabinets, personal protective equipment (PPE), and aerosol-minimizing techniques during pre-analytical phases, is important [81]. These considerations are crucial not only for laboratory safety but also for the scalability of saliva-based screening and diagnostics in resource-limited settings, where biosafety infrastructure may be minimal. Standardized protocols, risk assessments, and training are essential to ensure the safe implementation of salivary TB screening and diagnostics in both clinical and community settings.

## 5. Conclusions

Although salivary diagnostic techniques for tuberculosis are gaining traction due to their non-invasive nature, they remain far from fruition. Our systematic review and meta-analyses indicate that individual interleukins—such as IL-6, IL-5, and IL-17—do not consistently differentiate between patients with active TB and healthy or ORD controls. While IL-17 was found to be reduced in active TB, overall cytokine profiles were heterogeneous, and the data remain limited in scope and quantity. As for molecular diagnostic assays, GeneXpert assays performed significantly better than their counterparts but were not considered economically feasible as per WHO TPP guidelines.

Given the complexity of TB pathophysiology and the multitude of confounding factors that can influence cytokine expression, these salivary biomarkers may be more appropriate as tools for mass screening or interim monitoring during DOT regimens rather than for definitive diagnosis.

## Figures and Tables

**Table 1:** Eligibility criteria for (a) primary outcome and (b) secondary outcome.

(**a**)		
**PICO Component**	**Inclusion**	**Exclusion**
Population (P)	Adult humans (≥18 years old) with active TB or latent TB	Pediatric populations, animal populations, non-TB populations
Intervention (I)	Measurement of salivary interleukin levels	Studies without data on salivary interleukins
Comparison (C)	Measurement of salivary interleukin levels in controls (healthy/ORD)	Studies without comparable groups
Outcome (O)	Quantitative interleukin data (e.g., pg/mL) with diagnostic relevance (e.g., sensitivity, specificity)	Outcomes unrelated to diagnostic accuracy
Other Criteria	Diagnostic accuracy studies, including cross-sectional, prospective, or retrospective study design	Reviews, letters, personal opinions, book chapters, theses, conference posters or papers, patents, meta-analyses
	Studies in English	Non-English studies
	Moderate or low bias (QUADAS-2)	High risk of bias (QUADAS-2)
(**b**)		
**PICO Component**	**Inclusion**	**Exclusion**
Population (P)	Adult humans (≥18 years old) with active TB or latent TB	Pediatric populations, animal populations, non-TB populations
Intervention (I)	Salivary Mtb levels from molecular assays (e.g., GeneXpert MTB/RIF Ultra, GeneXpert MTB/RIF (Xpert), etc.) in active or latent TB patients	Unrelated interventions not using molecular assays
Comparison (C)	Salivary Mtb levels from molecular assays (e.g., GeneXpert MTB/RIF Ultra, GeneXpert MTB/RIF (Xpert), etc.) in controls	Studies without comparable groups
Outcome (O)	Diagnostic accuracy metrics (e.g., sensitivity, specificity, AUC, cost per test)	Lack of diagnostic performance metrics
Other Criteria	Diagnostic accuracy studies, including cross-sectional, prospective, or retrospective study design	Reviews, letters, personal opinions, book chapters, theses, conference posters or papers, patents, meta-analyses
	Studies in English	Non-English studies
	Moderate or low bias (QUADAS-2)	High risk of bias (QUADAS-2)

**Table 2:** Characteristics of the studies included in the meta-analysis.

Study	Intervention Group	Control Group	Active TB Sample Size	Latent TB Sample Size	Control Sample Size	Total Sample Size
Jacobs 2016 [21]	Active TB patients	ORD	32	-	72	104
Jacobs 2016.2 [25]	Active (confirmed or probable) TB patients	ORD	18	-	33	51
Phalane 2013 [24]	Active TB patients	No TB Disease (not specified as ORD or healthy)	11	-	27	38
Pradeep 2023 [23]	Active TB patients	ORD	40	-	40	80
Namuganga 2017 [26]	Active and latent TB patients	ORD	39	21	18	78
Estévez 2020 [22]	Active and latent TB patients	Healthy	28	27	42	97

**Table 3:** Levels of interleukins from the studies included in the meta-analysis in active TB, latent TB, and control (ORD, healthy) patients.

Study	Interleukins	Active TB Levels (pg/mL)	Latent TB Levels (pg/mL)	Control Levels (pg/mL)
Jacobs 2016 [21]	IL-6	0.8	-	1.4
	IL-8	36.5	-	68.3
Jacobs 2016.2 [25]	IL-1β	16.9	-	36.4
	IL-16	20.01	-	56.1
	IL-17A	13.8	-	6.1
	IL-23	0.3	-	0.0
Phalane 2013 [24]	IL-5	0.9	-	0.0
	IL-6	37.3	-	0.0
	IL-9	0.0	-	0.0
	IL-17	18.9	-	12.6
Pradeep 2023 [23]	IL-1β	12.44	-	8.95
	IL-2	55.43	-	39.04
	IL-5	75.75	-	95.28
	IL-6	14.46	-	12.28
	IL-16	1253.91	-	1382.62
	IL-17	11.7	-	10.76
Namuganga 2017 [26]	IL-2	0.0	-	0.0
	IL-5	1.6	-	1.6
	IL-6	5.11	-	4.8
Estévez 2020 [22]	IL-1α	1331	965	670.5
	IL-6	2.1	2.0	6.1
	IL-12p40	4.807	0	2.326

**Table 4:** Egger’s test results.

Parameter	Value
Model	Weighted regression with multiplicative dispersion
Predictor	Standard error
Test statistic (t)	1.2252
Degrees of freedom (df)	19
*p*-value	0.2335
Intercept (Limit as SE at 0, b)	−2.7160
95% Confidence Interval (CI)	−4.0350, −1.3969

**Table 5:** Community-based triaging chosen criteria and minimal requirements [12].

Criterion	Minimal Requirements
Goal and potential market	A test used during a patient’s first encounter with the health-care system to identify patients with any symptoms or risk factors for active pulmonary TB, including patients coinfected with HIV, those who do not have TB, and those in need of referral for further confirmatory testing
Pricing (of individual tests)	<USD 2.00
Diagnostic sensitivity	Overall sensitivity should be >90% when compared with the confirmatory test for pulmonary TB
Diagnostic specificity	Overall specificity should be >70% when compared with the confirmatory test

## Data Availability

No new data were created or analysed in this study. Data sharing does not apply to this article.

## Supplementary Materials

The following supporting information can be downloaded at: https://www.mdpi.com/article/10.3390/microorganisms13050973/s1, Table S1: QUADAS Quality Assessment; Table S2: Characteristics of studies that examine salivary biomarker levels in TB+ patients; Table S3: Characteristics of studies examining the use of different assays, and compared against WHO Triage target product profiles (TPP); Table S4: Leave-one-out Analysis. References [82,83,84,85,86,87] are cited in the supplementary materials.
