# Supplementary material for: The Role of Salivary Diagnostic Techniques in Screening for Active Pulmonary Tuberculosis: A Systematic Review and Meta-Analysis
Source: Microorganisms. 2025 Apr 24;13(5):973. doi: 10.3390/microorganisms13050973 (PMC12114365; doi:10.3390/microorganisms13050973)
Supplement: Supplementary file 1 [file microorganisms-13-00973-s001.zip › Tables S2-S4.pdf]

Supplementary Materials

Table S2: Characteristics of studies that examine salivary biomarker levels in TB+ patients

Study	Assay	Biomarkers Studies	Control Group	General Findings
Jacobs 2016	Luminex multiplex immunoassay	interferon (IFN)-γ, CXCL1(GRO), interleukin (IL)-1α, IL-1β, IL-2, IL-5, IL-6, IL-8, IL-9, IL-10, IL-13, IL-15, IL-17A, macrophage derived chemokine (MDC), tumour necrosis factor (TNF)-α, IFN-γ inducible protein (IP)-10, vascular endothelial growth factor (VEGF), monocyte chemotactic protein (MCP)-1, macrophage inflammatory protein (MIP)-1β, fractalkine, granzyme A, soluble Fas (sFas), soluble Fas Ligand (sFasL), solubleCD-137 (sCD137) (Merck Millipore, Billerica, MA, USA), and alpha-2-macroglobulin (A2M), haptoglobin, c-reactive protein (CRP), serum amyloid P (SAP), procalcitonin (PCT), ferritin, tissue plasminogen activator (TPA), fibrinogen and serum amyloid A (SAA)	ORD	A study of 104 individuals identified a seven-marker biosignature from saliva that diagnosed pulmonary TB with 78.1% sensitivity and 83.3% specificity; changes in several markers post-treatment may also help monitor response to TB therapy, but further validation is needed.
Jacobs 2016.2	Luminex Multiplex Immunoassay	(NCAM, transhyretin, MIP-4, antithrombin-III, GDF-15, ADAMTS13, (in kits purchased from Merck Millipore, Billerica, MA, USA), and other host markers namely: alpha2 macroglobulin (A2M), haptoglobin, C-reactive protein (CRP), serum amyloid P (SAP), procalcitonin (PCT), ferritin, tissue plasminogen activator (TPA), fibrinogen, serum amyloid A (SAA) (in kits purchased from Bio- Rad Laboratories, Hercules, CA, USA), vitronectin, extracellular matrix protein 1 (ECM1), vitamin D binding protein, sFas, granzyme A, sFasL, sCD137, granzyme_B, perforin, myoglobulin, P-selectin, lipocalin-2, thrombopoietin (TPO), stem cell factor (SCF), B-cell attracting chemokine 1 (BCA-1), epithelial neutrophil activating protein (ENA)-78, thymic stromal lymhpopoietin (TSLP), I-309(CCL-1), stromal cell derived factor 1 alpha (SDF-1α), IFN-γ, IFN-α 2, interferon inducible protein (IP)-10, macrophage inflammatory protein (MIP)-1β, tumor necrosis factor (TNF)-α, TNF-β, vascular endothelial growth factor (VEGF), soluble CD40 ligand (sCD40L), apolipoprotein (Apo) A-1, Apo CIII, complement component 3, complement factor H (CFH), total plasminogen activator inhibitor 1 (PAI-1), brain-derived neurotrophic factor (BDNF), cathepsin D, myeloperoxidase (MPO), matrix metalloproteinase (MMP)-2, MMP-9, hemofiltrate CC chemokine 1 (HCC-1), α-1-antitrypsin, pigment epithelium derived factor (PEDF), complement C4, interleukin (IL)-17F, IL-17A, IL-22, IL-33, IL-21, IL-23, IL-25, IL-31, IL-28A, IL-16, IL-1β, IL-12(p40) and IL-13	ORD	In a study of 51 participants, a five-marker salivary biosignature diagnosed TB disease with 88.9% sensitivity and 89.7% specificity, while an eight-marker biosignature achieved 100% sensitivity in non-HIV-infected individuals; changes in 11 markers during treatment suggest potential for monitoring treatment response, but further validation is needed.

Phalane 2013	Luminex Multiplex Immunoassay	interferon (IFN)-*γ*, interleukin (IL)-1*β*, IL-1*α*, IL-2, IL-4, IL-5, IL-6, IL-7, IL-8, IL-9, IL-10, IL-12(p70), IL-13, IL-15, IL-17, soluble IL-2 receptor alpha (sIL-2R*α*), interferon inducible protein (IP)-10, tumor necrosis factor (TNF)-*α*, fractalkine, granulocyte monocyte colony stimulating factor (GM-CSF), epidermal growth factor (EGF), monocyte chemotactic protein (MCP)-1, macrophage inflammatory protein (MIP)-1*β*, soluble CD40 ligand (sCD40L), transforming growth factor (TGF)-*α*, vascular endothelial growth factor (VEGF), granulocyte colony stimulating factor (G-CSF), CXCL1(GRO), C-reactive protein (CRP), serum amyloid protein A (SAA), serum amyloid protein P (SAP), matrix metalloproteinase (MMP)-2, and MMP-9	Healthy controls	A study of 38 individuals found that eight salivary inflammatory markers (IL-6, CRP, IL-9, IL-5, MIP-1β, fractalkine, IL-17, and VEGF) were significantly different between tuberculosis cases and non-cases, with all but VEGF showing higher median levels in TB patients; IL-6, CRP, MIP-1β, and fractalkine demonstrated potential diagnostic accuracy (AUC ≥ 0.70) despite no markers achieving sensitivity ≥64%, indicating that salivary markers could be valuable for diagnosing tuberculosis, especially in those unable to provide quality sputum samples.
Pradeep 2023	ELISA	IL-2,5,6,16,17 and 1β	ORD	In a study comparing interleukin (IL) levels in serum and saliva of pulmonary tuberculosis (PTB) patients, IL-2 and IL-17 were higher in serum, while IL-5 and IL-1β were higher in saliva; after two months of treatment, IL-2 and IL-16 levels decreased significantly in both samples, and IL-17 only in serum, indicating that IL-2, IL-16, and IL-17 may be useful for monitoring treatment response, though their diagnostic role in PTB remains unclear.
Namuganga 2017	Luminex Multiplex immunoassay	interferon gamma (IFN-γ), interleukin (IL) -2, 5, 6, granulocyte colony stimulating factor (G- CSF), granulocyte monocyte colony stimulating factor (GM-CSF), macrophage inflammatory protein (MIP)-1α and β, vascular endothelial growth factor (VEGF) and tumor necrosis factor alpha (TNF-α)	ORD	A study found higher IL-2 and IL-17 levels in serum and IL-5 and IL-1β in saliva of pulmonary tuberculosis (PTB) patients; IL-2, IL-16, and IL-17 may help monitor treatment response, though their diagnostic role is unclear. Additionally, a five-marker biosignature (serum IL-6, MIP-1β, VEGF, salivary G-CSF, and MIP-1α) showed 81.5% sensitivity and 100% specificity in diagnosing TB, while a three-marker salivary model had lower performance, indicating potential for biomarker combinations in diagnosis and monitoring.
Estevez 2020	Luminex Multiplex immunoassay	interferon (IFN)-γ and -α2, Tumor necrosis factor (TNF)-α and -β, IL-1α, IL-1β, IL-2, IL-3, IL-7, IL-12p40, IL-12p70, IL-15, IL-16, IL-17A IL-17F, IL-21, IL-22, IL-23, IL-32, IL-1Ra, IL-4, IL-5, IL-9, IL-10, IL-13, IL-6, IL-27, IL-33, the soluble form of the CD40 ligand (sCD40L), Transforming growth factor (TGF)-α, IL-8 (CXCL-8), B cell-attracting chemokine 1 (BCA-1 or CXCL-13), Eotaxin (CCL-11), Fibroblast Growth	Healthy controls	A study aimed at identifying host biomarkers to differentiate active tuberculosis (TB) patients from those with latent infection (LTBI) and uninfected individuals found that the best serum markers for distinguishing active TB from NoTBI contacts were [IP-10 + IL-7], while

		Factor-2 (FGF-2), FMS-related tyrosine kinase 3 Ligand (FLT-3L), Fractalkine (CX3CL1), GRO (CXCL1), interferon-inducible protein 10 (IP-10 or CXCL-10), Monocyte chemoattractant protein 1 (MCP-1 or CCL-2) and 3 (MCP-3 or CCL-7), Macrophage-derived chemokine (MDC or CCL-22), Macrophage inflammatory protein (MIP)-α and -β (or CCL-3 and CCL-4, respectively), RANTES (CCL5), Epidermal growth factor (EGF), Granulocyte-colony stimulating factor (G-CSF), Granulocyte-macrophage colony-stimulating factor (GM-CSF), Platelet-derived growth factor (PDGF)-AA and -AB/BB and the vascular endothelial growth factor (VEGF)		[Fractalkine + IP-10 + IL-1α + VEGF] in saliva also showed promise; the combination [IP-10 + BCA-1] in serum and IP-10 in saliva effectively distinguished active TB from LTBI, and markers like TNFα in serum and [Fractalkine + IL-12p40] in saliva differentiated NoTBI from LTBI, highlighting the potential of these biomarkers for TB diagnosis.

Table S3: Characteristics of studies examining the use of different assays, and compared against WHO Triage target product profiles (TPP)

References	Assay	Sensitivity (%) (95% CI)	Specificity (%) (95% CI)	AUC (95% CI)	p-value	**Meets minimal TPP for Triage**			
						Pricing	Diagnostic sensitivity	Diagnostic specificity	Goal and potential market
Meyer 2017 [20]	GeneXpert MTB/RIF (Xpert) on Saliva	66 (53–77)	96 (93–98)	0.81 (NR)	0.25	No (7.97 USD/unit)	No	Yes	Yes
Mesman 2019 [29]	GeneXpert MTB/RIF Ultra on Saliva	45 (29-62)	100 (89-100)	0.45 (NR)	NR	No (7.97 USD per unit)	No	Yes	Yes
Byanyima 2022 [30]	GeneXpert MTB/RIF Ultra on Saliva	90 (81-95)	NR	NR	<0.0001	No	Yes	N/A	Yes
Tang 2023 [82]	GeneXpert MTB/RIF (Xpert) on saliva	56.4 (49.7-62.9)	97.6 (95.5–98.8)	77.0 (72.7–81.3)	< 0.0001	No	No	Yes	Yes
Tang 2023 [82]	GeneXpert MTB/RIF Ultra on saliva	78.9 (72.8-83.8)	96.6 (94.3–98.1)	87.7 (84.4–91.1)	< 0.0001	No	No	Yes	Yes
Shi 2018 [28]	GeneXpert MTB/RIF (Xpert) on Saliva	38.07 (NR)	26.25 (NR)	NR	< 0.01	No	No	No	Yes
Shenai 2013 [83]	GeneXpert MTB/RIF (Xpert) on Saliva	38.5 (22.4-57.5)	NR	NR	< 0.001	No	No	N/A	Yes
Holani 2014 [84]	Auramine Rhodamine (AR) staining of salivary sample	76 (NR)	94 (NR)	NR	< 0.001	No	No	Yes	Yes
Raras 2014 [85]	Salivary sIg-A response against recombinant 38 kDa antigen	80 (67.82-92.18)	36.6 (21.92-51.27)	NR	0.01	NR	No	No	Yes
Raras 2024 [86]	Lateral flow assay using polyclonal antibody against Ag38 in saliva	100 (NR)	42.86 (NR)	NR	NR	NR	Yes	No	Yes
Hansen 2022 [87]	Lipobiotin-capture Magnetic Bead Assay on Saliva	70.6 (NR)	90 (NR)	NR	6.038205×10^{-35}	No	No	Yes	Yes

Table S4: Leave-one-out Analysis

Study	Estimate	SE	Z-value	P-value	CI Lower Bound (ci.lb)	CI Upper Bound (ci.ub)	Q	Qp	Tau²	I² (%)	H²
Jacobs 2016.1	-0.2859	0.6253	-0.4571	0.6476	-1.5115	0.9398	814.826	0	3.3929	94.3474	17.6909
Phalane 2013.1	-0.2518	0.6235	-0.4038	0.6864	-1.4739	0.9703	815.804	0	3.4032	94.3659	17.7492
Pradeep 2023.1	-0.1547	0.6799	-0.2275	0.82	-1.4871	1.1778	664.1658	0	3.7583	91.2844	11.4737
Estévez 2020.1	-0.4685	0.6176	-0.7587	0.448	-1.679	0.7419	803.6683	0	3.0829	93.7799	16.077
Jacobs 2016.2	0.2299	0.333	0.6905	0.4899	-0.4227	0.8826	38.6259	0.0049	**0.4612**	**53.0534**	**2.1301**
Phalane 2013.2	-0.256	0.6172	-0.4149	0.6782	-1.4656	0.9536	815.6987	0	3.3612	94.3022	17.5507
Pradeep 2023.2	-0.1628	0.665	-0.2448	0.8066	-1.4661	1.1406	814.1765	0	3.6722	94.6967	18.8562
Namuganga 2017.1	-0.2597	0.6634	-0.3914	0.6955	-1.56	1.0406	809.409	0	3.6365	94.6382	18.6503
Namuganga 2017.2	-0.2294	0.6782	-0.3382	0.7352	-1.5586	1.0998	721.3875	0	3.7392	93.5773	15.5697
Phalane 2013.3	-0.2278	0.6583	-0.346	0.7293	-1.518	1.0624	813.4212	0	3.6257	94.6555	18.7107
Pradeep 2023.3	-0.2252	0.6289	-0.3582	0.7202	-1.4579	1.0074	816.1178	0	3.4469	94.4312	17.9572
Pradeep 2023.4	-0.2604	0.6399	-0.4069	0.6841	-1.5145	0.9938	814.714	0	3.4985	94.4995	18.1801
Jacobs 2016.3	-0.2226	0.616	-0.3615	0.7178	-1.4299	0.9846	816.5372	0	3.3649	94.3092	17.5722
Pradeep 2023.5	-0.2262	0.6155	-0.3675	0.7133	-1.4326	0.9802	816.5239	0	3.3612	94.3035	17.5546
Estévez 2020.2	-0.2259	0.6151	-0.3673	0.7134	-1.4315	0.9797	816.5372	0	3.3587	94.2996	17.5427
Jacobs 2016.4	-0.2226	0.6161	-0.3613	0.7179	-1.4301	0.9849	816.5369	0	3.3657	94.3104	17.5759
Phalane 2013.4	-0.2274	0.6528	-0.3484	0.7276	-1.5069	1.0521	814.3603	0	3.5933	94.6225	18.5961
Estévez 2020.3	-0.1759	0.6413	-0.2743	0.7839	-1.4327	1.081	816.2374	0	3.5281	94.5442	18.3291
Jacobs 2016.5	-0.237	0.6181	-0.3835	0.7014	-1.4484	0.9744	816.2597	0	3.3747	94.3238	17.6173
Pradeep 2023.6	-0.226	0.6152	-0.3673	0.7134	-1.4317	0.9797	816.5356	0	3.359	94.3	17.544
Jacobs 2016.6	-0.3896	0.657	-0.5929	0.5533	-1.6773	0.8982	616.2487	0	3.4281	93.1103	14.5145
